# Candidate emotional awareness–interoceptive pathways in body-focused repetitive behaviors and somatic symptom disorder: a hypothesis-generating mini review

**DOI:** 10.3389/fpsyt.2026.1867350

**Published:** 2026-08-12

**Authors:** Yahui Zhang, Meijing Feng, Rui Ma, Qiuyan Xue, Xue Li, Fangming Liu

**Affiliations:** Department of Health Management, Aerospace Center Hospital, Beijing, China

**Keywords:** alexithymia, body-focused repetitive behaviors, emotional awareness, interoception, somatic symptom disorder, transdiagnostic

## Abstract

Body-focused repetitive behaviors (BFRBs) and somatic symptom disorder (SSD) are classified in different chapters in DSM-5. The former is characterized by repetitive body-focused actions, whereas the latter is characterized by distressing somatic symptoms accompanied by excessive thoughts, feelings, or behaviors related to those symptoms. Although parallel evidence has accumulated in each domain, direct comparative studies between BFRBs and SSD remain lacking. This gap arises partly from diagnostic separation, divergent research paradigms, and inconsistent terminology. The present mini review therefore proposes a hypothesis-generating conceptual framework in which difficulty identifying feelings and altered interoceptive processing may represent candidate processes relevant to both conditions. However, the strength of evidence is asymmetric. In SSD, interoceptive response bias has been examined using objective and signal-detection-based paradigms, whereas in BFRBs direct objective evidence remains limited. Accordingly, the proposed BFRB pathway should be regarded as a tentative hypothesis rather than an established mechanism. We further outline operational definitions, methodological considerations, and exploratory research questions for future comparative studies.

## 1 Introduction

A fifteen-year-old adolescent repeatedly picks the skin around the fingertips during examinations until bleeding occurs; when asked for the reason, no clear explanation is provided. A forty-two-year-old woman experiences persistent palpitations and chest tightness. Clinical assessment indicates benign premature ventricular contractions without evidence of severe cardiac disease, yet she repeatedly monitors her pulse, seeks reassurance from different physicians, worries excessively about bodily sensations, and avoids exercise for fear that the symptoms may worsen.

The former presentation is consistent with body-focused repetitive behaviors (BFRBs), whereas the latter is consistent with somatic symptom disorder (SSD). Importantly, SSD is not defined by the absence of a medical explanation. Rather, it is characterized by distressing somatic symptoms accompanied by excessive thoughts, feelings, or behaviors related to those symptoms. This distinction also separates SSD from illness anxiety disorder, in which health anxiety is prominent but somatic symptoms are absent or only mild. The two phenomena appear markedly different, yet both point to a possible shared process: when emotions cannot be clearly identified, poorly differentiated affective arousal may be encoded, monitored, or regulated through bodily sensations or repetitive body-focused behaviors.

In DSM-5, BFRBs are classified under “Obsessive-Compulsive and Related Disorders,” whereas SSD is classified under “Somatic Symptom and Related Disorders”. In the present article, the term BFRBs is used primarily to refer to DSM-5-recognized body-focused repetitive behavior disorders, particularly trichotillomania and excoriation disorder. These two conditions are emphasized because they have the clearest clinical and empirical links to repetitive body-focused urges, emotion regulation difficulties, and clinically significant impairment. Related behaviors such as onychophagia, dermatophagia, lip biting, or cheek biting are considered only as possible extensions of the proposed framework when they are clinically significant and body-focused, rather than as equivalent diagnostic entities. Accordingly, the proposed emotional awareness–interoceptive pathway is hypothesized to apply most directly to trichotillomania and excoriation disorder, whereas its relevance to other repetitive grooming behaviors remains more tentative and requires subtype-specific empirical testing.

This categorical separation has resulted in a longstanding lack of dialogue between the two fields: research on BFRBs focuses on behavioral reduction, whereas research on SSD emphasizes cognitive restructuring, with different terminologies and methodological tools employed in each domain. Such separation has prevented a key question from being raised: Could these two clinically distinct conditions share candidate psychological processes, such as emotional awareness deficits and interoceptive response bias?

Existing studies provide preliminary clues. In the BFRB literature, emotion regulation models have been established as a central framework (1), and pathological skin picking has been associated with broader emotion regulation difficulties and emotion reactivity (2). Epidemiological data indicate that the prevalence of BFRBs in the general population is approximately 1–3% (3, 4). In addition, a recent study of Turkish medical students reported that BFRBs were associated with depression, anxiety, stress, impulsivity, and difficulties in emotion regulation, suggesting that these behaviors may occur within a broader context of affective dysregulation and stress-related vulnerability (5). In the SSD field, alexithymia has been repeatedly shown to be associated with somatic symptoms (6, 7), and interoceptive response bias has been identified as a potentially important feature (8, 9). Placing the two conditions within a unified framework is therefore not intended to claim an established shared mechanism, but rather to generate a theoretically grounded and empirically testable hypothesis. At the phenomenological level, both conditions may involve bodily manifestations of poorly differentiated affective states; at the empirical level, both literatures implicate difficulty identifying feelings; and at the theoretical level, both may be linked through models of interoceptive processing and alexithymia. Together, these lines of evidence suggest a possible, but not yet confirmed, emotional awareness–interoceptive pathway. The present article integrates parallel evidence from both domains and proposes a heuristic framework for future comparative research.

### 1.1 Scope of review and literature search

This article is a narrative conceptual review rather than a systematic or scoping review. The literature was identified through targeted searches of PubMed/MEDLINE, PsycINFO, and Web of Science. Search terms included “body-focused repetitive behavior,” “BFRB,” “trichotillomania,” “excoriation disorder,” “somatic symptom disorder,” “SSD,” “alexithymia,” “difficulty identifying feelings,” “interoception,” “interoceptive accuracy,” “interoceptive bias,” “emotion regulation,” and “transdiagnostic.”

Studies were selected based on their relevance to emotional awareness deficits, interoceptive processing, BFRBs, SSD, and transdiagnostic models. Particular attention was given to studies addressing difficulty identifying feelings, interoceptive accuracy or response bias, bodily urges, somatic symptom monitoring, and emotion regulation. Because the aim of this article is to develop a theoretical framework rather than to provide an exhaustive synthesis of all available evidence, no formal systematic inclusion or exclusion procedure was applied. Accordingly, the proposed model should be understood as a hypothesis-generating framework for future empirical research rather than as a systematic review of the literature.

## 2 Parallel evidence in BFRB and SSD

### 2.1 Emotional awareness and alexithymia-related evidence: DIF as a candidate dimension

Because the available evidence differs in sample type and diagnostic level, we distinguish below between findings from diagnosed clinical samples and those from subclinical, community, or general-population samples. Findings from symptom-level or population-based studies are used to generate hypotheses rather than to establish disorder-level mechanisms. Alexithymia comprises three subdimensions: difficulty identifying feelings (DIF), difficulty describing feelings (DDF), and externally oriented thinking (EOT). In the BFRB field, evidence linking alexithymia-related constructs to trichotillomania remains preliminary and should be interpreted cautiously. In a clinical sample of patients with trichotillomania, Rufer et al. (10) reported that DIF predicted hair-pulling severity within the patient group. This finding supports an association between difficulty identifying feelings and symptom severity in trichotillomania, but it does not constitute evidence that patients with trichotillomania have elevated DIF scores compared with healthy controls. Moreover, the overall effect was modest, the association involving EOT was only marginal, and the authors called for prospective replication. Snorrason et al. (2) reported that pathological skin picking was associated with broader emotion regulation difficulties and heightened emotion reactivity, but the study did not assess alexithymia or TAS-20 subdimensions. Therefore, it should not be interpreted as direct evidence for DIF-specific emotional awareness deficits in excoriation disorder. In the SSD-related literature, De Gucht and Heiser (6) reported in a quantitative review that DIF showed a stronger association with somatic symptoms than DDF or EOT. However, this evidence primarily concerns somatic symptom burden and somatization rather than exclusively diagnosed SSD. Similarly, Mattila et al. (7) found in a general-population sample that DIF remained associated with somatization after controlling for somatic diseases and emotional disorders. Therefore, these findings are best interpreted as symptom-level or population-based evidence relevant to SSD, rather than direct evidence from diagnosed SSD samples.

Key observation: In both BFRBs and SSD, the relevant emotional-awareness difficulty appears to involve the early stage of emotional processing—identifying and labeling affective states. However, DIF should not be understood as specific to BFRBs or SSD. Rather, DIF is a nonspecific transdiagnostic vulnerability that has also been reported across depression, anxiety disorders, eating disorders, trauma-related disorders, obsessive-compulsive disorder, and personality pathology. Therefore, the proposed BFRB–SSD bridge does not lie in DIF alone. Instead, we hypothesize that DIF becomes clinically relevant to bodily expression when it interacts with interoceptive response bias and disorder-specific regulatory outputs. In BFRBs, poorly differentiated affective arousal may be experienced as bodily tension or urges and regulated through action-oriented repetitive behaviors such as pulling or picking. In SSD, poorly differentiated affective arousal may be interpreted through symptom monitoring, threat-based appraisal, reassurance seeking, and avoidance. Thus, DIF is proposed as an upstream vulnerability whose clinical expression depends on how bodily signals are interpreted and regulated, rather than as a mechanism specific to BFRBs or SSD.

### 2.2 Interoceptive characteristics: bias rather than reduced accuracy

In the BFRB field, O’Connor et al. (11) found that, prior to behavioral episodes, patients often report a vague bodily urge described as “not quite right,” and that discomfort subsides following the behavior. Teng et al. (12) reported that BFRB-related behaviors in a nonclinical, self-selected university sample were associated with higher self-reported somatic symptom frequency. Because the PILL cannot distinguish attentional bias from true symptom frequency, this finding should be interpreted as indirect evidence of somatic symptom reporting or interoceptive sensibility-like processes, rather than objective somatic perception in diagnosed BFRB. Objective assessment of interoception is typically conducted using heartbeat perception tasks (13). In recent years, interoception has been further differentiated into three dimensions: interoceptive accuracy, interoceptive sensibility, and interoceptive awareness (14). To avoid conceptual ambiguity, the key interoceptive terms used in this article are defined as follows. Interoceptive accuracy refers to the objective ability to detect internal bodily signals and can be assessed using heartbeat counting or heartbeat discrimination paradigms (13, 14). Interoceptive response bias, including a liberal response bias, refers to the tendency to report or interpret bodily signals as present or significant under uncertain sensory evidence; this can be examined using signal detection indices such as decision criterion, which separates response bias from perceptual sensitivity (8). Interoceptive sensibility refers to self-reported attention to, or perceived sensitivity toward, bodily sensations and can be assessed using validated self-report questionnaires such as the Multidimensional Assessment of Interoceptive Awareness or the Body Perception Questionnaire. Somatic perception refers to the conscious appraisal or interpretation of bodily sensations, particularly when such sensations are attributed to symptoms or bodily threat. Bodily urge refers to a subjective premonitory, tension-like, or “not-just-right” sensation that precedes repetitive body-focused behaviors. Finally, interoceptive deficit is used here only as an umbrella term and should be specified according to the affected domain, such as reduced accuracy, altered response bias, heightened sensibility, or abnormal appraisal. In the present model, the central candidate process is not a general interoceptive deficit, but a possible interoceptive response bias interacting with difficulty identifying feelings and disorder-specific regulatory outputs.

In the SSD field, debate regarding interoception has persisted for more than a decade. The “amplification hypothesis” posits that patients are hypersensitive to bodily signals, whereas the “deficit hypothesis” suggests impaired interoceptive ability. A meta-analysis by Wolters et al. (8), synthesizing 68 studies, concluded that interoceptive accuracy in patients with SSD is intact, but response bias is more liberal (r = −.163). The problem lies not in “perception,” but in “judgment”. Schaefer et al. (9) further found that greater severity of somatization is associated with lower interoceptive accuracy (r = −.44). Rief and Broadbent (15) proposed that individuals with alexithymia may misinterpret physiological arousal accompanying emotions as somatic symptoms.

### 2.3 Measurement operationalization of interoceptive bias and related constructs

To move the proposed framework from conceptual description to empirical testing, the key constructs should be operationalized using measurable, preferably non-self-report, indices. Interoceptive accuracy can be assessed using heartbeat counting or heartbeat discrimination paradigms, which quantify the objective ability to detect internal bodily signals. However, accuracy alone is insufficient to distinguish perceptual sensitivity from judgmental bias. Therefore, these paradigms should ideally be combined with signal detection approaches, in which indices such as sensitivity (*d′*) and decision criterion can be used to separate interoceptive accuracy from liberal or conservative response bias. In this framework, interoceptive response bias refers to the tendency to report or interpret bodily signals as present or significant under uncertain sensory evidence, rather than to heightened bodily perception per se.

In addition, psychophysiological indices may provide objective markers of bodily signal processing during emotional or symptom-relevant states. Heart rate variability, skin conductance responses, respiratory parameters, and autonomic reactivity during emotional challenge tasks could be used to quantify physiological arousal and regulatory flexibility. For BFRB, these measures may be collected before, during, and after urge episodes or experimentally induced stress. For SSD, they may be collected during symptom attention, bodily monitoring, or health-threat interpretation tasks. Combining behavioral interoceptive paradigms, signal detection modeling, and psychophysiological recordings would allow future studies to test whether BFRB and SSD share a liberal interoceptive response bias, differ in perceptual sensitivity, or show disorder-specific patterns of autonomic regulation.

### 2.4 Emotion regulation characteristics: preserved capacity and initiation difficulty

In the SSD field, systematic experimental evidence on emotion regulation has been established. In the BFRB field, although emotion regulation models serve as a central framework^[1]^, no experimental studies have yet distinguished between “capacity deficits” and “initiation difficulties”. The present section primarily reviews evidence from the SSD domain; the gap in the BFRB domain is addressed in Section 2.5 and future directions.

Schnabel et al. (16) found that patients with SSD report greater difficulties in emotion regulation (d = 1.62), whereas no differences are observed in regulatory performance on behavioral tasks compared with control groups. A key finding is that patients with SSD report higher levels of subjective effort (p = .038). The authors conclude that the problem lies “not in a deficit of capacity,” but rather in the fact that regulation is “too effortful to initiate”.

### 2.5 Comparison of parallel evidence and strength of evidence

The parallel evidence across emotional awareness, interoception, and emotion regulation in BFRB and SSD is summarized in **Table 1**. It should be noted that there is a marked asymmetry in the strength of the evidence described above. On the SSD side, conclusions regarding interoception are supported by a meta-analysis of 68 studies (8) as well as multiple experimental investigations (9, 16), and can therefore be considered relatively robust. In contrast, no studies in the BFRB field have employed objective interoceptive paradigms. The bodily urges reported by O’Connor et al. (11) merely indicate the presence of interoceptive signals. However, such findings cannot distinguish among three possibilities: “intact accuracy but biased responding,” as observed in SSD; “reduced accuracy,” as reported in eating disorders; or “heightened sensitivity,” as seen in panic disorder. Therefore, the present inference regarding interoceptive bias in BFRB should be regarded as a theoretical hypothesis rather than a definitive conclusion. In addition, the BFRB field lacks experimental studies that differentiate between “capacity deficits” and “initiation difficulties” in emotion regulation. These two gaps constitute critical targets for testing the proposed model.

**Table 1:** Parallel evidence in BFRB and SSD across emotional awareness, interoception, and emotion regulation.

Dimension	BFRB	SSD
Deficits in emotional awareness	TTM: DIF was associated with hair-pulling severity within the patient group, but no case-control elevation in DIF was established (10). Skin picking: evidence supports broader emotion regulation difficulties, not DIF-specific impairment (2).	DIF shows stronger associations with somatic symptoms than DDF or EOT (6, 7), but much of this evidence is symptom-level or population-based rather than exclusively diagnosed SSD.
Interoceptive characteristics	Bodily urges may precede repetitive behaviors (11). Teng et al. provides nonclinical self-report evidence of somatic symptom reporting/interoceptive sensibility-like processes, not objective somatic perception in diagnosed BFRB (12).	SSD evidence suggests relatively intact interoceptive accuracy but more liberal response bias (8, 9).
Emotion regulation characteristics	Emotion regulation models are central in BFRB (1), but experimental evidence separating capacity deficits from initiation difficulties is lacking.	Preserved regulation capacity but greater subjective effort/difficulty initiating regulation (16).

DIF, difficulty identifying feelings; DDF, difficulty describing feelings; EOT, externally oriented thinking. The cited studies differ in sample type and evidentiary level. Some findings are derived from diagnosed clinical samples, whereas others are based on subclinical, community, or general-population samples. Findings from nonclinical or symptom-level studies are used to generate hypotheses rather than to establish disorder-level equivalence between diagnosed BFRB and SSD.

Despite the asymmetry in evidence strength, the parallel findings outlined above are sufficient to support a preliminary integrative framework. The model proposed below should be considered a heuristic theoretical construction.

## 3 Integration: a candidate transdiagnostic framework

### 3.1 Theoretical bridge: alexithymia as a general deficit in interoception

Brewer et al. (17) demonstrated that individuals with alexithymia not only have difficulty identifying emotions but also show impaired discrimination between emotional and non-emotional interoceptive states (e.g., hunger and fatigue). This suggests that alexithymia may fundamentally reflect a general deficit in interoception. At the neural level, emotional and non-emotional interoceptive processes share common substrates, including the insular cortex and anterior cingulate cortex (18). Murphy et al. (19) further proposed that interoceptive deficits may represent a common vulnerability factor across multiple psychiatric disorders.

### 3.2 An integrative model within the predictive coding framework

The following model should be understood as a heuristic predictive-coding hypothesis rather than as an empirically established mechanism. Importantly, the evidentiary bases for the two pathways are not equivalent. The SSD pathway is supported by relatively stronger meta-analytic and experimental evidence on interoceptive accuracy, response bias, alexithymia, and emotion regulation. By contrast, the BFRB pathway currently relies more heavily on clinical observation, self-report findings, and indirect inference, because objective interoceptive measurements in BFRBs remain lacking. Accordingly, the BFRB component of the model should be regarded as a more tentative hypothesis requiring direct empirical testing. According to predictive coding theory (20), the brain continuously generates predictions about bodily states and compares them with incoming sensory signals. Mismatches generate “prediction errors,” which drive action to reduce the discrepancy. When the precision of prior beliefs is abnormally high, individuals rely more on beliefs than on sensory input when making judgments. This framework has been applied to explain the processing of interoceptive information (21, 22). Within this model, DIF is treated as a nonspecific upstream vulnerability rather than a disorder-specific mechanism. The more specific hypothesis is that DIF, when combined with interoceptive response bias and different regulatory action-selection patterns, may contribute to distinct bodily expressions in BFRBs and SSD.

More specifically, the proposed model can be mapped onto several predictive-coding parameters. In SSD, we hypothesize that illness-related prior precision may be elevated, whereas sensory precision may be relatively preserved. Ambiguous bodily sensations may therefore be weighted through strong health-threat priors and interpreted as medically significant. This profile may be accompanied by a liberal interoceptive decision criterion, in which bodily signals are more readily judged as meaningful or threatening under uncertain sensory evidence. Prediction errors may then be resolved through symptom monitoring, reassurance seeking, avoidance, and repeated medical consultation.

**Figure 1:**
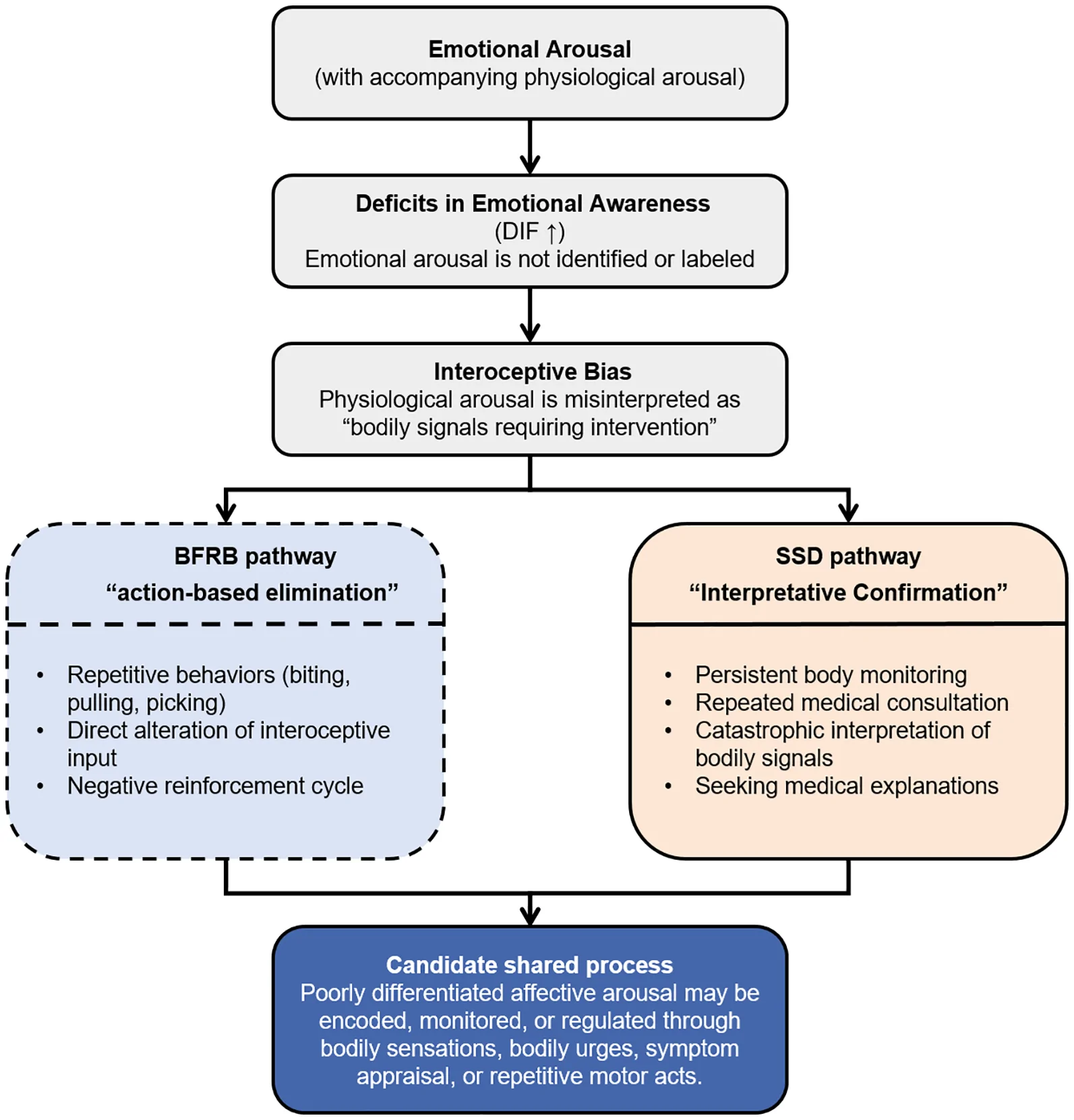
A heuristic transdiagnostic framework of candidate emotional awareness–interoceptive pathways in BFRBs and SSD. Solid elements indicate processes with relatively stronger empirical support, particularly in the SSD literature. Dashed elements indicate more tentative components, especially the proposed interoceptive response-bias pathway in BFRBs, which currently lacks direct evidence from objective interoceptive paradigms. The model also distinguishes candidate predictive-coding parameters across conditions: SSD is hypothesized to involve stronger illness-related prior precision, relatively preserved sensory precision, a liberal interoceptive decision criterion, and threat-based regulatory outputs, whereas BFRBs are more tentatively hypothesized to involve bodily urge- or “not-just-right”-related priors, altered prediction-error weighting under poorly differentiated affective states, and action-oriented regulatory outputs. This model is intended to guide future empirical testing and should not be interpreted as a confirmed shared mechanism or as evidence that BFRBs and SSD currently rest on equivalent evidentiary footing.

For BFRBs, the predictive-coding profile is more tentative because objective interoceptive evidence remains limited. We hypothesize that some patients may show elevated precision of bodily urge-related or “not-just-right” priors, together with a liberal decision criterion for bodily tension or urge signals. Under conditions of poorly differentiated affective arousal, prediction-error weighting may shift toward action-oriented regulation, such that pulling, picking, or other repetitive body-focused behaviors are selected to reduce bodily tension or uncertainty. Thus, BFRBs and SSD are not assumed to share identical predictive-coding profiles. Rather, they may show partially overlapping interoceptive response bias, while differing in prior content, prediction-error interpretation, and regulatory action selection. The hypothesized transdiagnostic pathway and the unequal evidentiary footing of the SSD and BFRB components are summarized in **Figure 1**.

Emotional awareness deficits deprive individuals of an “emotional template” for interpreting emotionally induced bodily sensations. Interoceptive signals therefore fail to be matched to specific emotional categories and are experienced as prediction errors with unclear origin. In SSD, relatively stronger evidence suggests that such ambiguity may contribute to symptom monitoring, threat interpretation, reassurance seeking, and avoidance. In BFRBs, a parallel but more tentative pathway is hypothesized, in which poorly differentiated affective arousal may be experienced as bodily tension or urges and regulated through repetitive body-focused actions. Thus, the proposed candidate shared process is not that emotions are symbolically “expressed through the body,” but that unidentified or poorly differentiated affective arousal may be encoded, monitored, or regulated through bodily sensations, bodily urges, symptom-related appraisal, or repetitive motor acts. In this view, the primary impairment lies in identifying and labeling affective states, rather than in somatic registration itself.

Within this framework, the “initiation difficulty” reported by Schnabel et al. (16) can be interpreted as follows: failure to recognize emotional states leads to poorly specified regulatory goals, thereby increasing subjective cognitive effort and ultimately reducing the likelihood of initiating emotion regulation in daily life.

It should be emphasized that no objective interoceptive measurement evidence is currently available in the BFRB literature. Accordingly, the proposed mechanisms of interoceptive bias in BFRB should be regarded as theoretical hypotheses rather than established findings.

### 3.3 Speculative extension: state-dependent and temporal dynamics

The proposed emotional awareness–interoceptive pathway may also be examined as a state-dependent process rather than a fixed trait-like mechanism. This extension remains speculative because direct longitudinal and ecological momentary evidence comparing BFRBs and SSD is currently lacking. Future studies could test whether difficulty identifying feelings and interoceptive response bias become more clinically relevant during periods of heightened affective arousal, stress exposure, bodily vigilance, or reduced reflective processing. In BFRBs, such states may precede bodily tension, urges, and repetitive behaviors; in SSD, they may precede symptom monitoring, threat interpretation, reassurance seeking, and avoidance. Ecological momentary assessment combined with psychophysiological sampling may help determine when these candidate processes become clinically active.

### 3.4 Speculative extension: contextual and structural factors

A further speculative extension is that emotional awareness and interoceptive processing may be shaped by broader contextual factors, including chronic stress, cultural norms regarding emotional expression, healthcare access, gender socialization, and socioeconomic adversity. These factors may influence both the intensity of bodily arousal and the ways in which poorly differentiated affective states are interpreted or regulated. Because direct evidence linking these contextual factors to the proposed BFRB–SSD pathway is limited, they are presented here only as possible moderators for future research rather than as established components of the model.

## 4 Clinical implications and future directions

### 4.1 Clinical implications

First-line treatment for BFRBs is habit reversal training (HRT) (23), whereas SSD is commonly treated with cognitive behavioral therapy (CBT) (24). The present model does not imply that these established interventions are insufficient, nor does it suggest that emotional awareness training has already been shown to improve treatment outcomes in either condition. Rather, it generates testable intervention hypotheses for future research.

Specifically, future studies could examine whether adding emotional awareness-focused components to HRT may benefit patients with BFRBs who show elevated difficulty identifying feelings, particularly when repetitive behaviors are preceded by poorly differentiated bodily tension, discomfort, or urges. Similarly, studies in SSD could test whether integrating emotional awareness work into CBT may improve outcomes in patients whose symptom monitoring, reassurance seeking, or health-related worry is strongly linked to poorly differentiated affective arousal.

These hypotheses could draw on existing approaches that target emotion identification, labeling, and regulation, including the emotion regulation skills module in dialectical behavior therapy (DBT) (25) or emotion regulation therapy (ERT) (26). Mindfulness- or body-awareness-based interventions may also be relevant because they explicitly train attention to bodily signals while encouraging non-catastrophic interpretation of interoceptive experience. Bornemann and Singer (27) demonstrated that a 9-month meditation training program can improve interoceptive accuracy and reduce alexithymia, while Farb et al. (28) proposed that contemplative practice may shift bodily processing from active inference toward sensory inference. However, whether such approaches can improve HRT outcomes in BFRBs or CBT outcomes in SSD remains an empirical question. Therefore, emotional awareness training should be considered a potential additional mechanistic target for selected patients rather than an established treatment recommendation.

### 4.2 Study limitations and future directions

This review is limited by the lack of studies directly comparing BFRB and SSD. Two major gaps in the BFRB literature—the absence of objective interoceptive measurement and the lack of experimental studies on emotion regulation—remain to be addressed. Therefore, the proposed BFRB–SSD pathway should be regarded as a heuristic framework for future empirical testing rather than an established transdiagnostic mechanism.

A transdiagnostic perspective, such as the RDoC framework, emphasizes moving beyond traditional diagnostic categories and focusing on shared dimensions of psychopathology (29). Interoceptive deficits have been proposed as a common vulnerability factor across multiple psychiatric disorders (30). However, the specificity of the present model also requires empirical validation. Interoceptive profiles may differ fundamentally across disorders: Panic disorder is characterized by heightened sensitivity, whereas eating disorders are characterized by reduced accuracy. BFRB and SSD may share a profile of relatively intact interoceptive accuracy but abnormal response bias, although this remains to be directly tested, particularly in BFRB. Whether this pattern is specific to these two disorders or represents a broader feature of the internalizing spectrum remains unclear. Future comparative studies could examine whether panic disorder, eating disorders, BFRB, and SSD show distinct profiles of interoceptive sensitivity and response bias, rather than assuming that BFRB and SSD necessarily share the same interoceptive profile.

Another important direction concerns the state-dependent and temporal dynamics of the proposed pathway. Emotional awareness deficits and interoceptive response bias may not operate as fixed trait-like processes, but may become particularly salient during periods of heightened emotional arousal, limited reflective processing, stress exposure, or increased bodily monitoring. In BFRB, this may involve a pre-urge escalation cycle in which poorly differentiated affective arousal is translated into bodily tension or urges, followed by repetitive behavior as short-term regulation. In SSD, a parallel cycle may involve bodily sensation detection, threat interpretation, symptom monitoring, and further amplification of distress. These dynamic processes may help explain why symptoms fluctuate across contexts and why specific regulatory windows may be especially important for intervention.

Given the absence of direct comparative studies between BFRB and SSD, future research should begin with exploratory comparisons rather than assuming equivalence between the two clinical groups. Three open research questions may guide this work:

RQ1: Do patients with BFRB and patients with SSD show elevated DIF scores compared with healthy controls, and does the magnitude or clinical role of DIF differ between the two clinical groups?

RQ2: Do BFRB and SSD show similar or distinct interoceptive profiles when perceptual sensitivity and response bias are separated using signal detection indices?

RQ3: Does interoceptive response bias mediate the association between DIF and symptom severity within each disorder, and is this mediation pattern shared or disorder-specific?

In addition, future studies could test whether these associations are strengthened during high-arousal, high-stress, or high-bodily-monitoring states.

Future research may proceed in a staged manner. First, the profiles of DIF, interoceptive accuracy, and response bias should be compared between the two groups using objective interoceptive paradigms and signal detection indices. Second, ecological momentary assessment combined with repeated psychophysiological sampling should be used to examine the temporal chain of affective arousal, emotional clarity, bodily urge or symptom monitoring, and regulatory behavior in daily life. Measures such as heart rate variability, skin conductance responses, respiratory parameters, or autonomic reactivity during emotional challenge tasks may help determine whether interoceptive bias emerges specifically during state-dependent periods of emotional or bodily salience. Finally, the transdiagnostic efficacy of interventions targeting emotional awareness should be examined.

## 5 Conclusion

In DSM-5, BFRBs and SSD are classified under separate chapters; however, the parallel evidence reviewed in this article suggests that the two conditions may be usefully examined within a candidate emotional awareness–interoceptive framework. In this framework, difficulty identifying affective states may increase reliance on bodily signals, while interoceptive response bias and disorder-specific regulatory outputs may shape whether poorly differentiated arousal is monitored as somatic symptoms or regulated through repetitive body-focused behaviors. Nevertheless, direct comparative studies between BFRBs and SSD are currently absent. Therefore, the proposed model should be understood as a heuristic framework for future research rather than an empirically established pathway or confirmed shared mechanism. The value of the model lies in synthesizing parallel literatures, generating testable predictions, and encouraging future studies using objective interoceptive paradigms, signal detection approaches, ecological momentary assessment, and psychophysiological measures. If supported, this framework may suggest process-level commonalities across DSM categories; if not supported, it will still help define the first direct empirical comparisons between BFRBs and SSD. Thus, the present article should be viewed as a hypothesis-generating conceptual synthesis rather than evidence that the DSM categorical separation has already obscured a confirmed shared mechanism.

## References

[1] Roberts S O'Connor K Bélanger C . Emotion regulation and other psychological models for body-focused repetitive behaviors. Clin Psychol Rev. (2013) 33(6):745–762. doi: 10.1016/j.cpr.2013.05.004 23792470

[2] Snorrason I Smári J Olafsson RP . Emotion regulation in pathological skin picking: findings from a non-treatment seeking sample. J Behav Ther Exp Psychiatry. (2010) 41(3):238–45. doi: 10.1016/j.jbtep.2010.01.009 20172501

[3] Grant JE Chamberlain SR . Prevalence of skin picking (excoriation) disorder. J Psychiatr Res. (2020) 130:57–60. doi: 10.1016/j.jpsychires.2020.06.033 32781374 PMC7115927

[4] Houghton DC Maas J Twohig MP Saunders SM Compton SN Neal-Barnett AM . Comorbidity and quality of life in adults with hair pulling disorder. Psychiatry Res. (2016) 239:12–9. doi: 10.1016/j.psychres.2016.02.063 27137957 PMC4855296

[5] Korkmaz ŞA . Prevalence and influencing factors of body-focused repetitive behaviors in Turkish medical students. Genel Tıp Dergisi. (2024) 34:681–9. doi: 10.54005/geneltip.1505724

[6] De Gucht V Heiser W . Alexithymia and somatisation: A quantitative review. J Psychosom Res. (2003) 54:425–34. doi: 10.1016/S0022-3999(02)00467-1 12726898

[7] Mattila AK Kronholm E Jula A Salminen JK Koivisto AM Mielonen RL . Alexithymia and somatization in general population. Psychosom Med. (2008) 70(6):716–22. doi: 10.1097/PSY.0b013e31816ffc39 18596251

[8] Wolters C Gerlach AL Pohl A . Interoceptive accuracy and bias in somatic symptom disorder. PloS One. (2022) 17:e0271717. doi: 10.1371/journal.pone.0271717 35980959 PMC9387777

[9] Schaefer M Egloff B Witthöft M . Is interoceptive awareness really altered in somatoform disorders? J Abnorm Psychol. (2012) 121:719–24. doi: 10.1037/a0028509 22642840

[10] Rufer M Bamert T Klaghofer R Moritz S Schilling L Weidt S . Trichotillomania and emotion regulation. Psychiatry Res. (2014) 218 (1-2):161–5. doi: 10.1016/j.psychres.2014.03.029 24768249

[11] O'Connor K Brisebois H Brault M Robillard S Loiselle J. Behavioral activity associated with onset in chronic tic and habit disorder. Behav Res Ther. (2003) 41(2):241–249. doi: 10.1016/S0005-7967(02)00051-7 12547383

[12] Teng EJ Woods DW Twohig MP Marcks BA . Body-focused repetitive behavior problems: prevalence in a nonreferred population and differences in perceived somatic activity. Behav Modif. (2002) 26(3):340–60. doi: 10.1177/0145445502026003003 12080905

[13] Schandry R . Heart beat perception and emotional experience. Psychophysiology. (1981) 18:483–8. doi: 10.1111/j.1469-8986.1981.tb02486.x 7267933

[14] Garfinkel SN Seth AK Barrett AB Suzuki K Critchley HD . Knowing your own heart: Distinguishing interoceptive accuracy from interoceptive awareness. Biol Psychol. (2015) 104:65–74. doi: 10.1016/j.biopsycho.2014.11.004 25451381

[15] Rief W Broadbent E . Explaining medically unexplained symptoms. Clin Psychol Rev. (2007) 27:821–41. doi: 10.1016/j.cpr.2007.07.005 17716793

[16] Schnabel K Schulz SM Witthöft M . Emotional reactivity, emotion regulation, and regulatory choice in SSD. Psychosom Med. (2022) 84:1077–86. doi: 10.1097/PSY.0000000000001118 35980776

[17] Brewer R Cook R Bird G . Alexithymia: A general deficit of interoception. R Soc Open Sci. (2016) 3:150664. doi: 10.1098/rsos.150664 27853532 PMC5098957

[18] Khalsa SS Adolphs R Cameron OG Critchley HD Davenport PW Feinstein JS . Interoception and mental health: A roadmap. Biol Psychiatry Cognit Neurosci Neuroimaging. (2018) 3:501–13. doi: 10.1016/j.bpsc.2017.12.004 29884281 PMC6054486

[19] Murphy J Brewer R Catmur C Bird G . Interoception and psychopathology: a developmental neuroscience perspective. Dev Cognit Neurosci. (2017) 23:45–56. doi: 10.1016/j.dcn.2016.12.006 28081519 PMC6987654

[20] Paulus MP Stein MB . Interoception in anxiety and depression. Brain Struct Funct. (2010) 214:451–63. doi: 10.1007/s00429-010-0258-9 20490545 PMC2886901

[21] Seth AK Suzuki K Critchley HD . An interoceptive predictive coding model of conscious presence. Front Psychol. (2011) 2:395. doi: 10.3389/fpsyg.2011.00395 22291673 PMC3254200

[22] Barrett LF Simmons WK . Interoceptive predictions in the brain. Nat Rev Neurosci. (2015) 16:419–29. doi: 10.1038/nrn3950 26016744 PMC4731102

[23] Woods DW Houghton DC . Evidence-based psychosocial treatments for pediatric BFRBs. J Clin Child Adolesc Psychol. (2016) 45:227–40. doi: 10.1080/15374416.2015.1055860 26167847

[24] Kleinstäuber M Witthöft M Hiller W . Efficacy of short-term psychotherapy for multiple MUPS. Clin Psychol Rev. (2011) 31:146–60. doi: 10.1016/j.cpr.2010.09.001 20920834

[25] Linehan MM . Cognitive-Behavioral Treatment of Borderline Personality Disorder. New York: Guilford Press (1993).

[26] Mennin DS Holaway RM Fresco DM Moore MT Heimberg RG . Delineating components of emotion and its dysregulation in anxiety and mood psychopathology. Behav Ther. (2007) 38(3):284–302. doi: 10.1016/j.beth.2006.09.001 17697853

[27] Bornemann B Singer T . Taking time to feel our body. Psychophysiology. (2017) 54:469–82. doi: 10.1111/psyp.12790 27925645

[28] Farb N Daubenmier J Price CJ Gard T Kerr C Dunn BD . Interoception, contemplative practice, and health. Front Psychol. (2015) 6:763. doi: 10.3389/fpsyg.2015.00763 26106345 PMC4460802

[29] Insel T Cuthbert B Garvey M Heinssen R Pine DS Quinn K . Research domain criteria (RDoC): Toward a new classification framework for research on mental disorders. Am J Psychiatry. (2010) 167(7):748–51. doi: 10.1176/appi.ajp.2010.09091379 20595427

[30] Caspi A Houts RM Belsky DW Goldman-Mellor SJ Harrington H Israel S . The p factor: One general psychopathology factor in the structure of psychiatric disorders? Clin Psychol Sci. (2014) 2(2):119–37. doi: 10.1177/2167702613497473 25360393 PMC4209412

